# Lycopene Attenuates Tulathromycin and Diclofenac Sodium-Induced Cardiotoxicity in Mice

**DOI:** 10.3390/ijms19020344

**Published:** 2018-01-24

**Authors:** Mohamed M. Abdel-Daim, Rasha Eltaysh, Azza Hassan, Shaker A. Mousa

**Affiliations:** 1 Pharmacology Department, Faculty of Veterinary Medicine, Suez Canal University, Ismailia 41522, Egypt; 2 Department of Pharmacology, Faculty of Veterinary Medicine, Mansoura University, Mansoura 35516, Egypt; rasha_mans@yahoo.com; 3 Pathology Department, Faculty of Veterinary Medicine, Cairo University, Giza 12122, Egypt; azzahassan99@gmail.com; 4 Pharmaceutical Research Institute, Albany College of Pharmacy and Health Sciences, New York, NY 12144, USA; shaker.mousa@acphs.edu

**Keywords:** cardiotoxicity, diclofenac sodium, lycopene, mice, tulathromycin

## Abstract

Recent experiments showed a potential cardiotoxic effect of the macrolide antibiotic (tulathromycin). This study was performed to investigate whether diclofenac sodium (DFS) potentiates the cardiotoxicity of tulathromycin and increases the cardioprotective effects of lycopene against DFS and tulathromycin. Seven groups (eight per group) of adult Swiss albino mice received saline (control), tulathromycin (a single subcutaneous dose of 28 mg/kg/bw on day 14), DFS (a single oral dose of 100 mg/kg/bw on day 14), tulathromycin plus DFS, or lycopene (oral, 10 mg/kg/bw daily for 15 d) combined with tulathromycin, DFS, or both. Compared to the control group, the administration of tulathromycin or DFS (individually or in combination) caused significantly elevated (*p* < 0.05) serum levels of Creatine kinase-myocardial B fraction (CK-MB), lactate dehydrogenase, and cardiac-specific troponin-T and tissue levels of nitric oxide and malondialdehyde that were accompanied by significantly decreased tissue reduced glutathione content and glutathione peroxidase, superoxide dismutase, and catalase antioxidant enzyme activity. Upon histopathological and immunohistochemical examination, the mean pathology scores and the percentages of caspase-3-, Bax-, and CK-positive regions were significantly higher in the tulathromycin- and/or DFS-treated groups than in control mice. For all these parameters, the pathological changes were more significant in the tulathromycin–DFS combination group than in mice treated with either drug individually. Interestingly, co-administration of lycopene with tulathromycin and/or DFS significantly ameliorated the changes described above. In conclusion, DFS could potentiate the cardiotoxic effects of tulathromycin, whereas lycopene can serve as a cardioprotective agent against DFS and tulathromycin.

## 1. Introduction

Macrolide antibiotics (e.g., clarithromycin, azithromycin, and roxithromycin) are general bacteriostatic agents that are effective in the treatment of respiratory and soft tissue infections, because they are retained in tissues for several days following administration of a single dose of the drug [1,2]. However, their use at high doses can induce oxidative stress, increase mitochondrial membrane permeability, and precipitate arrhythmogenic events [3,4,5]. Tulathromycin is an effective macrolide that is used for the treatment of Gram-positive respiratory infections in cattle and swine [6,7]. Recent experiments showed that tulathromycin may exert cardiotoxic effects by increasing the production of reactive oxygen species (ROS) and altering serum levels of coagulation factors, potassium, and ionized calcium [8,9].

Diclofenac sodium (DFS) is a non-steroidal anti-inflammatory drug (NSAID) that inhibits the cyclo-oxygenase II (COX-II) enzyme, thereby blocking the production of pro-inflammatory prostaglandins [10]. Several studies have suggested that combining antibiotics with NSAIDs improves their efficacy by controlling pathogens and ameliorating the resulting inflammation [11,12]. However, prolonged DFS intake can exert cardiotoxic effects, such as increasing the risk of myocardial infarction and initiating or worsening congestive heart failure [13,14]. Moreover, a recent study showed that DFS augments the cardiotoxic effects of tilmicosin (TIL; another commonly used macrolide) by enhancing TIL-induced oxidative stress, and increasing the expression of the apoptotic enzymes caspase-3 and Bcl-2 [15]. Whether the same effect occurs when combining DFS and tulathromycin remains to be investigated.

Phytochemicals are plant-derived small molecules that possess antioxidant, anti-inflammatory, antimicrobial, and cardioprotective effects [16,17,18,19]. Among these compounds, lycopene is an acyclic carotenoid (a derivative of vitamin A) that can efficiently quench singlet oxygen (with twice the activity of β-carotene) [20], and displays strong free-radical scavenging activity (13.3 times higher than that of α-tocopherol) [21]. Moreover, lycopene exerts anti-inflammatory, immunostimulant, antibiotic, and antimutagenic effects [22]. Several epidemiological studies have reported that lycopene administration reduces serum low-density lipoprotein (LDL) cholesterol, arterial stiffness, and the risk of cardiovascular diseases (CVD) [23,24,25]. Further, in vivo experiments have shown that lycopene can protect against the cardiotoxicity induced by xenobiotics, such as doxorubicin [16,26,27], isoprotenol [28], and atrazine [29].

In light of these findings, this study was conducted to investigate whether DFS potentiates the cardiotoxic effects of tulathromycin, and whether lycopene can protect cardiac tissue against the cardiotoxicity caused by DFS and tulathromycin.

## 2. Results

### 2.1. Biochemical Analysis

**Figure 1:**
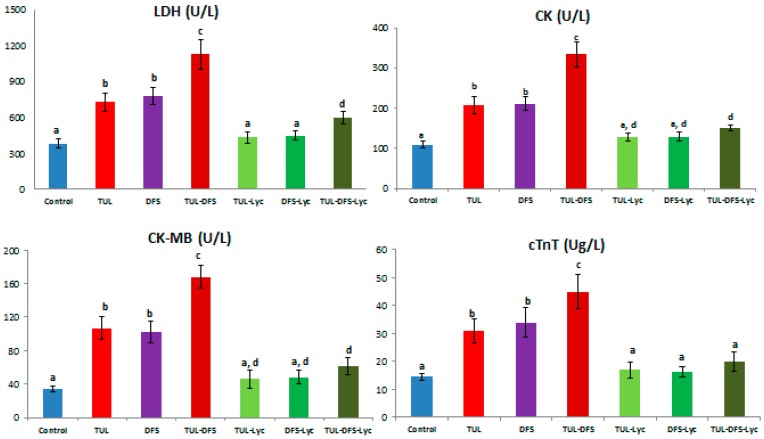
The effect of lycopene treatment on serum levels of cardiac injury biomarkers in tulathromycin and diclofenac sodium intoxicated rats. Data are means ± SD. Means carrying different superscripts (a,b,c,d) are significantly different at (*p* < 0.05). Abbreviations: CK: Creatine kinase, cTnT: Cardiac-specific troponin-T, DFS: Diclofenac sodium, LDH: Lactate Dehydrogenase, Lyc: Lycopene, TLR: Tulathromycin.

Biochemical serum analyses showed that serum levels of LDH, CK, CK-MB, and CTnT were higher in tulathromycin or DFS-treated mice, compared to the control group. When both agents (tulathromycin and DFS) were co-administered, significant elevations were observed relative to the control group for all of the serum components listed above. Tulathromycin- or DFS-administered mice that also received lycopene showed significant ameliorations of the elevated serum levels, restoring the concentrations of these components to their normal values. In tulathromycin–DFS-treated mice that also received lycopene, serum levels of all the aforementioned parameters were reduced, but only the CTnT level was restored to normal (Figure 1). 

**Figure 2:**
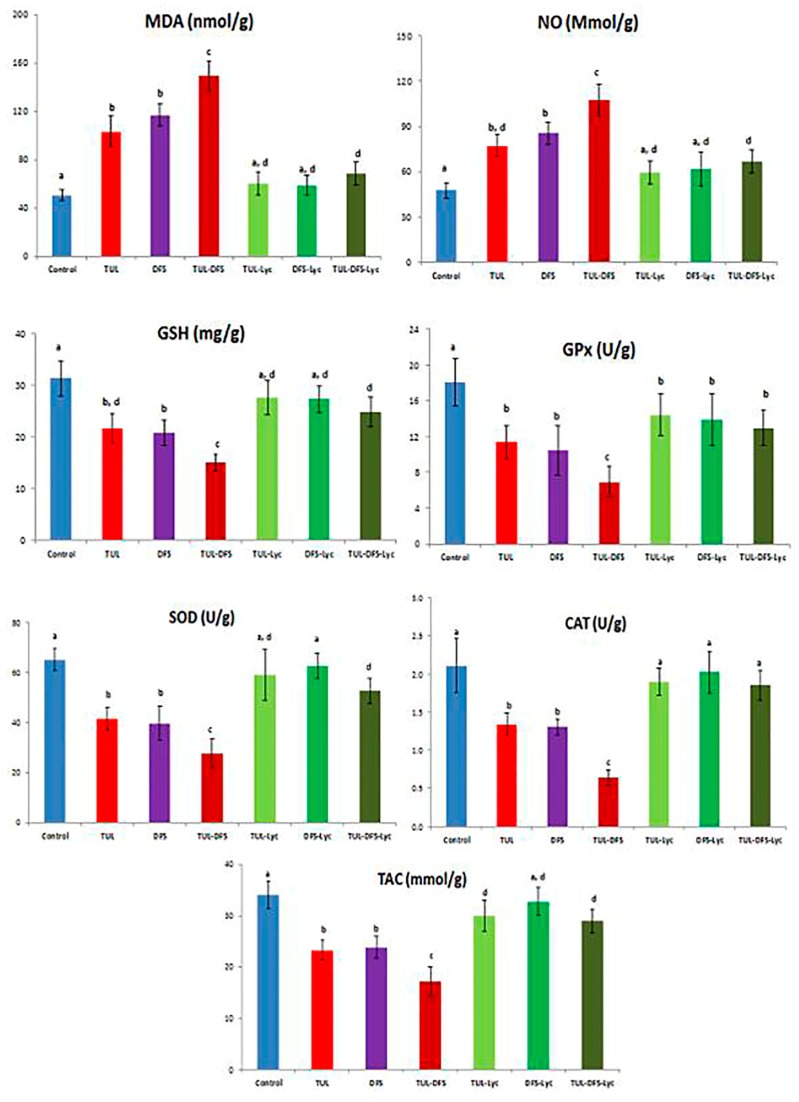
The effect of lycopene treatment on tissue lipid peroxidation and activities of antioxidant enzymes in tulathromycin and diclofenac sodium intoxicated rats. Data are means ± Standard deviation (SD). Means carrying different superscripts (a,b,c,d) are significantly different at (*p* < 0.05). Abbreviations: CAT: Catalase, DFS: Diclofenac Sodium, GSH: Reduced glutathione, GPx: Glutathione peroxidase, Lyc: Lycopene, MDA: Malondialdehyde, NO: Nitric oxide, SOD: Superoxide dismutase, TAC: Total antioxidant capacity, TLR: Tulathromycin.

Biochemical tissue analyses showed that tulathromycin- or DFS-treated mice had significantly higher cardiac tissue levels of MDA and NO, compared to the control group. Moreover, tulathromycin or DFS treatments were associated with significantly lower cardiac tissue GSH concentration, TAC, and activities of the GPx, SOD, and CAT enzymes. These changes were significantly amplified in mice receiving both tulathromycin and DFS. Treatment of tulathromycin or DFS-administered mice with lycopene restored the levels of all mentioned parameters to normal, except for TAC in tulathromycin-injected mice (which was elevated, but remained significantly lower than the control group) and GPx in both groups (in which no significant elevations were observed). In tulathromycin–DFS-treated mice that also received lycopene, pathological alterations in all the aforementioned tissue parameters were ameliorated, but only the CAT enzyme activity was restored to normal (Figure 2).

### 2.2. Histopathology

**Figure 3:**
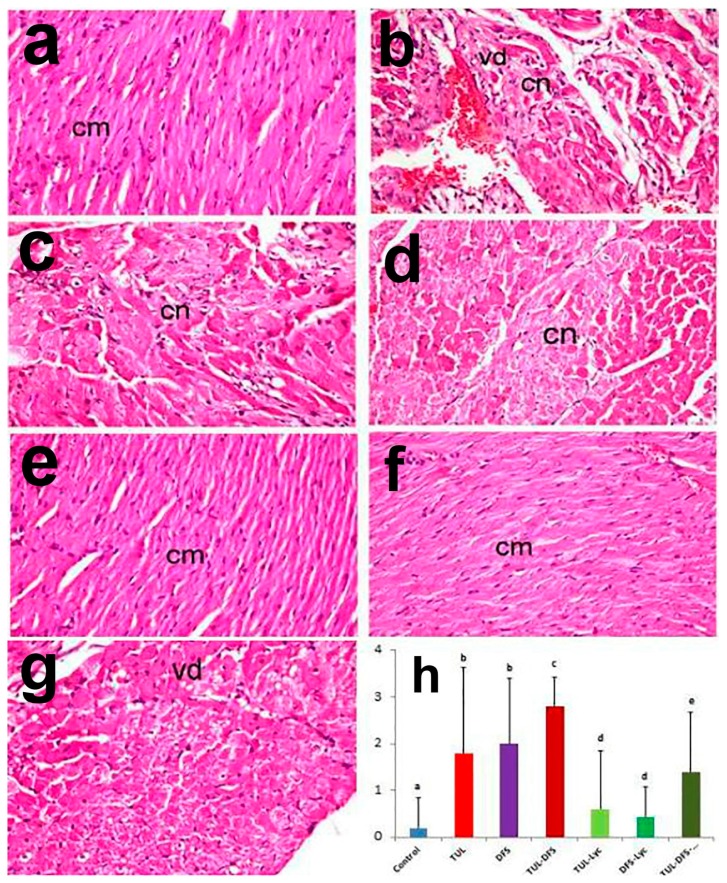
Light photomicrographs of heart tissue in (**a**) control mice showing normal architecture of cardiac muscle fibers (cm); (**b**) tulathromycin-treated mice showing extensive vacuolar degeneration (vd) associated with coagulative necrosis (cn) of cardiac myocytes in addition to apoptotic changes; (**c**) DFS-treated mice showing coagulative necrosis of cardiomyocytes (cn) with loss of cross-striation in cardiac muscle fibers; (**d**) tulathromycin–DFS-treated mice showing cardiomyocyte necrosis (cn), apoptosis and myocytolysis; (**e**) tulathromycin–lycopene-treated mice showing normal cardiac myocytes (cm); (**f**) DFS–lycopene-treated mice showing marked restoration of cardiomyocytes (cm); (**g**) tulathromycin–DFS–lycopene-treated mice showing mild vacuolar degeneration of cardiomyocytes (vd) (H&E, ×400); and (**h**) pathologic scoring of cardiotoxicity in the heart tissue of control and treated mice. Means carrying different superscripts (a,b,c,d,e) are significantly different at (*p* < 0.05).

Cardiac tissue sections from control mice showed normal histological architecture, with no identifiable degenerative, necrotic, or apoptotic cells (Figure 3a). By contrast, tissue sections from tulathromycin-injected mice revealed extensive macrophage infiltration and vacuolar degeneration that were associated with eosinophilic coagulative necrosis of cardiac myocytes and apoptotic changes (Figure 3b). The necrotic myocytes exhibited intense cytoplasmic eosinophilia that was associated with nuclear pyknosis or even karyolysis. Similarly, tissue sections from DFS-treated mice showed histopathological alterations that were similar to those seen in the tulathromycin-treated group. The main lesions were coagulative necrosis of cardiomyocytes (especially in the endocardium and myocardium), in addition to apoptotic changes and loss of cross-striations of some cardiac muscle fibers (Figure 3c). Additional tissue damage was observed in mice treated with both tulathromycin and DFS, in which extensive cardiomyocyte necrosis, apoptosis, myomalacia, and myocytolysis were noted, in addition to intense inflammatory cellular infiltration (Figure 3d). 

Marked amelioration of histopathological alterations was demonstrated in the tulathromycin–lycopene- and DFS–lycopene-treated groups, with restoration of cardiac myocytes that appeared relatively similar to the control group (Figure 3e,f). Moreover, regression of the histopathological lesions was demonstrated in the tulathromycin–DFS–lycopene-treated group, with mild vacuolar degeneration of cardiomyocytes (Figure 3g). Figure 3h shows that lesion scores were significantly higher in tulathromycin and/or DFS treated mice, compared to the control group. Significant ameliorations of lesion scores were noted in all lycopene treated mice, with restoration of normal scores in groups receiving tulathromycin or DFS, individually.

### 2.3. Immunohistochemistry

**Figure 4:**
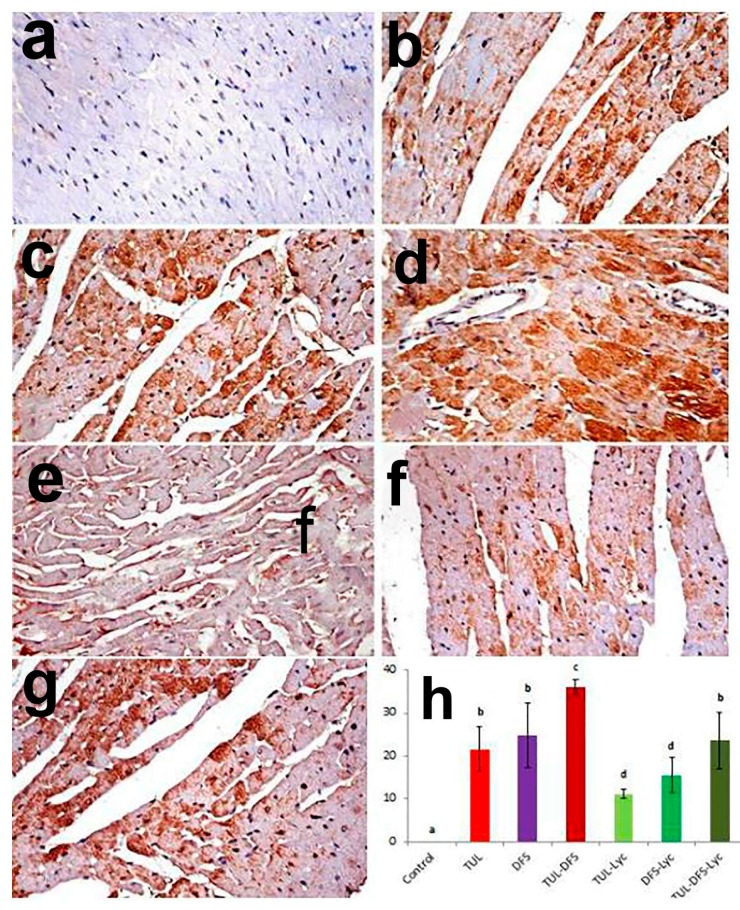
Staining for caspase-3 protein in a cardiac tissue section of (**a**) control mice showing no caspase-3 immune-reactive cells; (**b**) tulathromycin-treated mice showing multiple caspase-3 immune-reactive cells; (**c**) DFS-treated mice showing abundant caspase-3 immune-reactive cells; (**d**) tulathromycin–DFS-treated mice showing diffuse, intensely stained caspase-3 immune-reactive cells; (**e**) tulathromycin–lycopene-treated mice showing fewer caspase-3 immune-reactive cells; (**f**) DFD–lycopene-treated mice showing sparse caspase-3 immune-reactive cells; (**g**) tulathromycin–DFS–lycopene-treated mice showing sporadic caspase-3 immune-reactive cells (caspase-3 immunohistochemical staining, ×400); and (**h**) immunohistochemical scoring of caspase-3 in the heart tissue of control and treated mice. Means carrying different superscripts (a,b,c,d) are significantly different at (*p* < 0.05).

**Figure 5:**
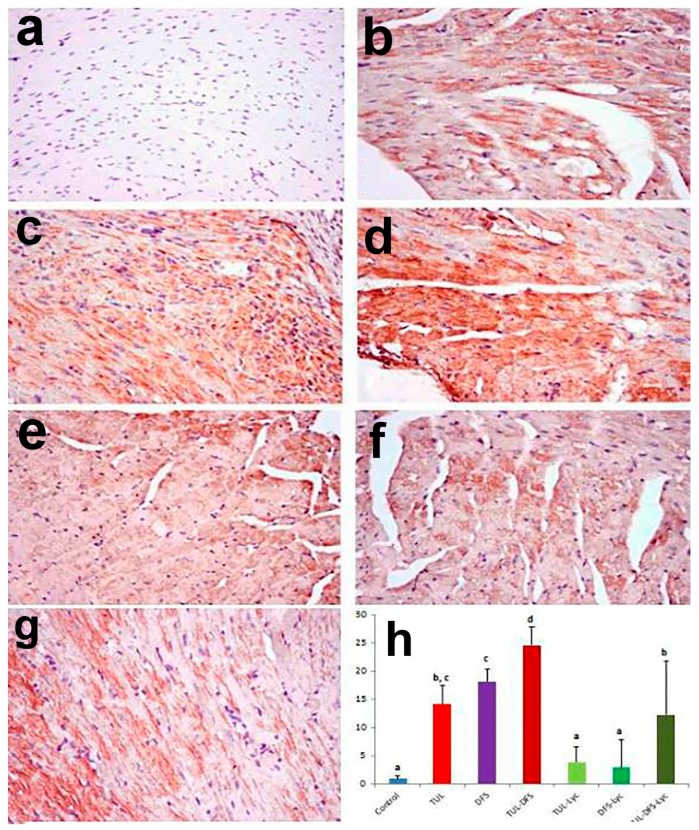
Staining for Bax protein in a section of heart tissue in (**a**) control mice showing no Bax immune-reactive cells; (**b**) tulathromycin-treated mice showing an increased number of Bax immune-reactive cells; (**c**) DFS-treated mice showing numerous Bax immune-reactive cells; (**d**) tulathromycin–DFS-treated mice showing intense staining of Bax immune-reactive cells; (**e**) tulathromycin–lycopene-treated mice showing fewer Bax immune-reactive cells; (**f**) DFS–lycopene-treated mice showing sparse Bax immune-reactive cells; (**g**) tulathromycin–DFS–lycopene-treated mice showing a reduced number of Bax immune-reactive cells (Bax immunohistochemical staining, ×400); and (**h**) immunohistochemical scoring of Bax protein in the heart tissue of control and treated mice. Means carrying different superscripts (a,b,c,d) are significantly different at (*p* < 0.05).

**Figure 6:**
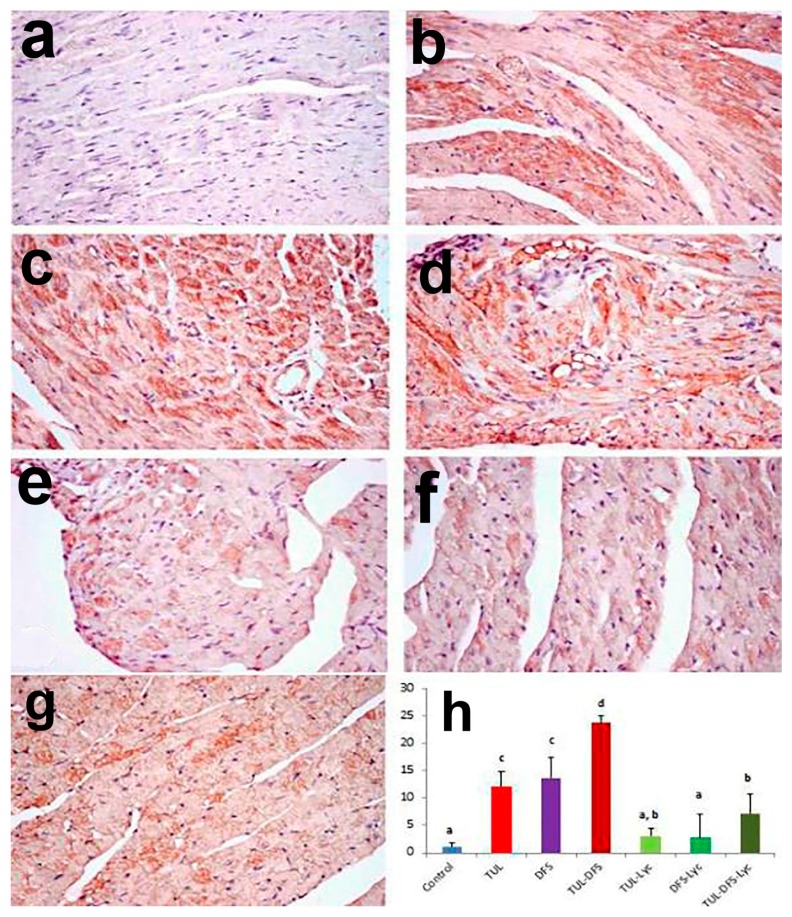
Staining for CK protein in a section of heart tissue in (**a**) control mice showing no CK immune-reactive cells; (**b**) tulathromycin-treated mice showing an increased number of CK immune-reactive cells; (**c**) DFS-treated mice showing abundant CK immune-reactive cells; (**d**) tulathromycin–DFS-treated mice showing numerous CK immune-reactive cells; (**e**) tulathromycin–lycopene-treated mice showing few individual CK immune-reactive cells; (**f**) DFS–lycopene-treated mice showing sparse CK immune-reactive cells; (**g**) tulathromycin–DFS–lycopene-treated mice showing sporadic CK immune-reactive cells (CK immunohistochemical staining, ×400); and (**h**) immunohistochemical scoring of Bax protein in the heart tissue of control and treated mice. Means carrying different superscripts (a,b,c,d) are significantly different at (*p* < 0.05).

Immunohistochemistry showed no caspase-3, Bax, or CK-immune-reactive cells in the cardiac tissue of control mice (Figure 4a, Figure 5a and Figure 6a, respectively). By contrast, an intensely positive caspase-3, Bax, and CK immune staining was seen in the tulathromycin- and DFS-treated groups (Figure 4b,c, respectively for caspase-3, Figure 5b,c, respectively for Bax, and Figure 6b,c, respectively for CK). Moreover, significantly higher expression levels of the caspase-3, Bax, and CK proteins were recorded in mice of the tulathromycin–DFS-treated group, compared to those in mice injected with tulathromycin or DFS individually (Figure 4d, Figure 5d and Figure 6d, respectively). 

On the other hand, weak caspase-3, Bax, and CK expression were recorded in the tulathromycin–lycopene-treated group (Figure 4e, Figure 5e and Figure 6e, respectively) and in the DFS–lycopene-treated group (Figure 4f, Figure 5f and Figure 6f, respectively). Moreover, reductions in caspase-3, Bax, and CK expression were recorded in the tulathromycin–DFS–lycopene-treated group relative to the tulathromycin–DFS-treated group (Figure 4g, Figure 5g and Figure 6g, respectively). Figure 4h, Figure 5h and Figure 6h summarizes the immunohistochemical evaluations of caspase-3, Bax, and CK protein expression in cardiac tissue sections from the control and treated mice.

## 3. Discussion

This study shows, for the first time, that DFS can potentiate the cardiotoxic effects of tulathromycin, and that lycopene can serve as a cardioprotective agent against tulathromycin. The potentiated cardiotoxic effects manifested as increased serum levels of cardiac injury biomarkers (CK-MB and CTnT), which may result from increased membrane leakage of necrotizing cardiomyocytes, and from increased lipid peroxidation and exhaustion of cellular antioxidant defense mechanisms (e.g., reduced GSH concentration and reduced GPx, SOD, and CAT enzyme activities). The cardiotoxic effects of both tulathromycin [8,9] and DFS [15,30] have been confirmed separately in previous studies.

An intriguing feature of macrolide antibiotics is their varied effects on oxidative status/lipid peroxidation in different studies. For example, erythromycin, azithromycin, roxithromycin and clarithromycin can reduce erythrocyte MDA levels in guinea pigs with experimental otitis media [31]. Moreover, clarithromycin (at low doses) was shown to increase GSH levels in cardiac tissue after exposure to doxorubicin [32]. On the other hand, tilmicosin [15] and tulathromycin [9] were shown to increase the serum and cardiac tissue levels of MDA, respectively. Er et al. suggested that these differences may be attributable to the different molecular structures of these macrolides, or the different dosing regimens in published studies [9]. However, further comparative studies are needed.

Previous studies have shown that DFS can increase ROS generation in the heart by the myocardial mitochondria, and by the NADPH oxidase enzyme in phagocytic cells [33] and cardiomyocytes [34]. Li and colleagues confirmed that DFS is a potent inducer of NADPH oxidase, thereby causing excessive ROS production [35]. Excessive ROS then alters cellular homeostasis, leading to the subsequent damage of such cellular components, such as lipids, proteins, nucleic acids, and the mitochondria themselves [36]. Other authors have reported that DFS can potentiate the oxidative stress induced by tilmicosin [15] and doxorubicin [37].

Histopathological examination revealed cardiac lesions in both the tulathromycin- and DFS-treated groups, with the most severe lesions seen in the combination group. These lesions consisted mainly of vacuolar degeneration and/or necrosis of cardiac myocytes, in addition to apoptotic changes. Although apoptosis was previously reported as a possible mechanism for the cardiotoxicity of macrolides (tilmicosin) [15], our study is, to the best of our knowledge, the first to show myocardial apoptosis as a possible mechanism of tulathromycin cardiotoxicity. In contrast, although several studies have shown that COX-II inhibitors can induce apoptosis, the involvement of COX-II in macrolide cardiotoxicity remains a matter of debate [38,39]. Moreover, Deavall et al. and Hickey et al. showed that oxidation of mitochondrial DNA by pro-oxidant xenobiotics can activate proapoptotic pathways and ultimately cause cell death [40,41]. 

As with other macrolides, a feared complication of tulathromycin is arrhythmias. Clarithromycin, in particular, has been shown in several studies to prolong the QT interval, and to induce torsades de pointes arrhythmias. This effect of clarithromycin was explained by its mitochondrial toxicity and blockade of potassium channels [3,4,5]. In a study by Er and colleagues, tulathromycin administration reduced serum levels of potassium and ionized calcium, pointing toward a possible mechanism of cardiotoxicity [8].

The results of the present study confirm the cardioprotective effect of lycopene, which can be attributed to its powerful antioxidant activity. Lycopene is one of the most commonly investigated phytochemicals in the literature, and current evidence confirms its utility in reducing the risk of CVD [42]. The protective effects of lycopene against tulathromycin cardiotoxicity in this study may be attributed to its mitigation of oxidative/nitrative stress (by enhancing the activities of cellular antioxidant enzymes, scavenging oxygen free radicals, and inhibiting the production of nitric oxide) and apoptosis (by inhibiting caspase-3 and Bax expression). Moreover, former studies showed that the anti-inflammatory effect of lycopene is mediated by inhibiting the nuclear factor-κB pathway and reducing the production of pro-inflammatory cytokines, such as the tumor necrosis factor-α and interleukins [43,44].

Previous in vivo/in vitro experiments examining lycopene cardioprotection against other xenobiotics suggested that it works by inhibiting the endothelial–monocyte interaction [45], suppressing the mRNA expression of fibrotic markers as transforming growth factor-β and collagen I/III [44], and inhibiting the activity of 3-hydroxy-3-methylglutaryl-coenzyme-A (HMG-CoA) reductase (the rate-limiting enzyme in cholesterol synthesis) [46]. These findings were further confirmed in interventional studies in the literature [47,48]. For example, Kim et al. investigated the efficacy of lycopene (15 mg/kg in tomato oleoresin capsules) in a randomized, placebo-controlled trial on 126 healthy men. They found that lycopene administration reduced oxidative stress and inflammatory biomarkers, and improved the endothelial function [48]. 

In light of our findings, future studies are encouraged to test the benefit of combining tulathromycin with DFS in animal models with microbial inflammation, and to weigh the benefits against the risks of cardiotoxicity. If further evidence regarding the cardiotoxicity of a combination of DFS and tulathromycin is obtained, future studies should outline necessary precautions and dosing regimens to avoid this potential adverse event. Further awareness about such cardiotoxicity should also be raised among practicing veterinarians. In conclusion, DFS can potentiate the cardiotoxic effect of tulathromycin, while lycopene can serve as a cardioprotective agent when used in combination treatment with DFS and tulathromycin.

In the current study, the effects of single DFS (100 mg/kg/bw) and tulathromycin (28 mg/kg/bw) administration on the heart were evaluated when given each alone or in combination. The limitation of this study is the use of single dose, and it is recommended to use DFS in different doses and frequencies to evaluate which dose and regimen will induce potentiated cardiotoxicity, and which could be protective against tulathromycin-induced cardiac injury. Further knowledge of molecular mechanisms will be required to evaluate all possible mechanisms involved in DFS and tulathromycin-induced cardiotoxicity.

## 4. Materials and Methods

### 4.1. Chemicals

Tulathromycin was administered as an injectable solution (Draxxin^®^, 100 mg∙mL^−1^ vial) obtained from animal health division of Pfizer Co. (Cairo, Egypt). Diclofenac sodium was obtained from Novartis Pharma Co. (Basel, Switzerland), while lycopene was purchased from Sigma Aldrich Co. (Saint Louis, MO, USA). Other solutions used for biochemical analyses were of analytical grade. All of the kits that were used were purchased from Biodiagnostics Co. (Cairo, Egypt), except for the kits to analyze the lactate dehydrogenase (LDH) (Randox Laboratories, UK), creatine kinase (CK) and creatine kinase-MB (CK-MB) enzymes (Stanbio, TX, USA) and to evaluate cardiac-specific troponin-T (cTnT) (Roche Diagnostics, Mannheim, Germany).

### 4.2. Animals and Experimental Procedures

Fifty-six male Swiss albino mice (22 to 27 g) were purchased from the Egyptian Organization for Biological Products and Vaccines. Mice were acclimatized for 7 days prior to the experiment at 25 ± 1 °C and normal humidity under a 12 h light/dark cycle. The experimental protocol was approved by the research ethics committee of the Faculty of Veterinary Medicine, Suez Canal University, Ismailia, Egypt (Approval No. 201708).

Mice were equally divided into seven groups, which received the following: (1) saline only (negative control); (2) a single subcutaneous dose of tulathromycin (28 mg/kg/bw) on the 14th day of the experiment; (3) a single oral dose of DFS (100 mg/kg/bw) on the 14th day; (4) tulathromycin and DFS (same doses and routes); (5) tulathromycin (same regimen as group 2) plus lycopene (10 mg/kg/bw, once daily, orally for 15 days); (6) DFS (same regimen as group 3) plus lycopene (same regimen as group 5); or (7) tulathromycin plus DFS (same regimens as groups 2 and 3, respectively) and lycopene (same regimen as group 5). After 24 h of tulathromycin or DFS administration, mice were sacrificed by cervical decapitation under isoflurane anesthesia, and blood samples were obtained in non-heparinized tubes. The clotted samples were then centrifuged at 3000 rpm for 20 min, and the supernatant sera were stored at −20 °C for later use. A portion of the cardiac tissue from each mouse was homogenized in 5 mL of phosphate buffer and centrifuged at 3000 rpm for 15 min at 4 °C for biochemical analyses.

### 4.3. Biochemical Analysis

The stored serum samples were used to evaluate levels of LDH [49], CK [50], CK-MB [51], and cTnT (using ELISA kits according to the manufacturer’s protocol, Roche Diagnostics, Germany). The homogenized cardiac tissue samples were used to measure cardiac tissue concentrations of malondialdehyde (MDA) [52], nitric oxide (NO) [53], and reduced glutathione (GSH) [54], total antioxidant capacity (TAC) [55], and the activities of the glutathione peroxidase (GPx) [56], superoxide dismutase (SOD) [57], and catalase (CAT) [58] enzymes. Each analysis was conducted once. 

### 4.4. Histopathological Examination

Different cardiac sections from control and treated mice were excised and fixed in 10% neutral formalin, and subsequently embedded in paraffin wax. Next, 4 µm-thick sections were stained with hematoxylin and eosin (H&E) and examined by light microscopy to assess histopathological lesions. Ten microscopic fields per section were examined. Semi-quantitative scoring was performed to analyze the lesions using the following parameters: 0 = tissue appeared normal; 1 = mild (up to 30% of the examined area was affected); 2 = moderate (31–60% of the examined area was affected); and 3 = severe (˃60% of the examined area was affected). The histopathological parameters used for evaluation included vacuolar degeneration and/or coagulative necrosis of cardiac myocytes, apoptosis, and inflammatory cell infiltration. 

### 4.5. Immunohistochemical Analysis

Caspase-3, Bax, and CK expression levels in cardiac sections were examined according to the method by Martín-Burriel et al. [59]. Sections were incubated with primary antibodies against caspase-3 (1:100 dilution), Bax (1:100 dilution), and CK (1:50 dilution) (Santa Cruz Biotechnology Inc., Dallas, TX, USA). The immune reaction was visualized using diaminobenzidine tetrachloride (DAB, Sigma Chemical Co., St. Louis, MO, USA). The positive immune reactive cells showed brown-stained cytoplasm and/or nuclei. The caspase-3, Bax, and CK protein expression was estimated by quantification of the threshold area for immunohistochemical brown color according to the method Castrogiovanni et al. [60].

### 4.6. Statistical Analysis

All values were normally distributed, and were expressed as the mean ± standard deviation from the mean (SD). To determine whether there was a statistically significant difference among experimental groups, the one-way analysis of variance (ANOVA) was used, followed by post hoc Tukey’s test. A *p* value of less than 0.05 was considered statistically significant. All statistical analyses were performed using the Statistical Package for Social Sciences (SPSS, Chicago, IL, USA, version 22 for Windows). 

## Abbreviations

CAT	Catalase
CK-MB	Creatine kinase-myocardial B fraction
CTnT	Cardiac-specific troponin-T
DFS	Diclofenac sodium
GSH	Reduced glutathione
LDH	Lactate dehydrogenase
MDA	Malondialdehyde
SOD	Superoxide dismutase
TAC	Total antioxidant capacity
