# Expression of Concern: Abdel-Daim et al. Lycopene Attenuates Tulathromycin and Diclofenac Sodium-Induced Cardiotoxicity in Mice. *Int. J. Mol. Sci.* 2018, *19*, 344

**DOI:** 10.3390/ijms27146168

**Published:** 2026-07-10

**Authors:** International Journal of Molecular Sciences Editorial Office

**Affiliations:** MDPI, St. Alban-Anlage 66, 4052 Basel, Switzerland; ijms@mdpi.com

With this notice, the IJMS Editorial Office wishes to alert readers to concerns regarding potentially duplicated areas in the lower-left corners of the images presented in Figures 3–6 of this article [1]. An evaluation was conducted by the Editorial Board; however, as the authors and affiliated institutions have not responded to the Editorial Office’s requests, these concerns could not be conclusively resolved. This notice is issued to ensure transparency and to maintain the integrity of the scholarly record. No further editorial action is planned at this time. The journal will reconsider the matter should additional information become available.
